# Anent “the Breeders’ Gazette’s” Attitude toward the Veterinary Profession

**Published:** 1902-12

**Authors:** 


### EDITORIALS.

### ANENT "THE BREEDERS' GAZETTE'S" ATTITUDE TOWARD THE VETERINARY PROFESSION.

If the editor of the *Breeders' Gazette* were asked whether his paper was engaged in a systematic effort to discredit and injure the veterinary profession, he might say that it was not. He might say that he was a great admirer of the profession and that he desired to help it so far as he could. Perhaps he would say that by pointing out items of veterinary work and effort that are in his estimation defective he is doing as a fond parent would do with an erring child.

Criticism may be friendly or it may be unfriendly. It may be for the purpose of helping or for the purpose of hurting. Whether it is the one or the other may be shown by its truthfulness and its form. If the criticism is based on false premises and contains falsehoods, if it is sweeping, if it contains innuendoes calculated to cause the impression that the situation is far worse than stated, and if it is expressed in harsh, derogatory language, it is safe to assume that it is uttered with unfriendly intent.

The above is general in its application, but we suggest that our readers use the standard laid down as a scale of points for judging the intent of the editors of the *Breeders' Gazette* when they write upon things veterinary.

In the *Gazette* for August 27, 1902, in an editorial on "Britain and the Argentine Ports," the following occurs: " . . . The altogether extraordinary circumstances which marked the closing of the Argentine ports have made British breeders very restive under the embargo, and certainly constitute one of the most inexplicable chapters in the history of veterinary blundering that has resulted in loss to the live-stock trade. The British ship "Highland Scot" last May embarked at Liverpool a large cargo of cattle and sheep for Buenos Ayres, and on arrival at port the official veterinarian announced that he discovered the presence of foot-and-mouth disease. The ship was immediately ordered out into the roads and quarantine established. The petition of the owners of the ship and of the stock for inspection of a jury of six veterinarians was granted, and their unanimous verdict was that the 'foot-and-mouth' scourge was not present. A few of the cattle were found afflicted with sore mouths, and this symptom, not uncommon, had been erroneously diagnosed by the official veterinarian. This is not surprising ; the astonishing feature of the affair is that, so far as present advices go, the wrong perpetrated by the blunder of an incompetent veterinarian has not been righted, and the trade of two countries has suffered in consequence. Little wonder is it that British breeders are harsh in their expressions on the present situation. The temper of breeders has already been so sorely taxed by veterinary authorities in many ways that too great caution cannot be exercised by them in the exercise of authority calculated to disturb internal or international trade."

Here is the *Breeders' Gazette* trying to load upon the veterinary profession the responsibility for an act that, it must be evident from its own testimony, cannot justly be placed upon the shoulders of any veterinarian. The facts of the matter are that foot-and-mouth disease existed in the Argentine Republic several years ago, and that cattle and sheep suffering with this disease were carried from the Argentine Republic to England, and still had the disease in a transmissible stage, for they conveyed it to cattle from the United States that they were brought into direct contact with at Deptford. When this occurred the British Government forbade the admission of live cattle from the Argentine Republic. British ships were sent down the channel to meet incoming cattle boats from the Argentine Republic that had sailed after the quarantine was declared, and it was required that they should slaughter and throw overboard all cattle and disinfect their boats before they were permitted to approach an English port. The people of the Argentine Republic felt badly about this, and upon the first opportunity retaliated by forbidding the admission of English cattle, upon the alleged ground that they were afflicted with the foot-and-mouth disease. It was entirely true that there was foot-and-mouth disease in England at that time, and it was quite possible that the English cattle on the "Highland Scot" were suffering from this disease. American cattle landed in England in 1881 and 1882 with foot-and-mouth disease when there was not a single case in this country, and it was proven that they were infected upon shipboard, probably by the use of head-ropes that had been used upon sick cattle. The English cattle that were sent to the Argentine Republic may have become infected in a similar way. The Argentine Republic has worked most strenuously to repress foot-and-mouth disease, and the government has just offered a law, copied from the English Contagious Diseases of Animals Acts, that is planned to make it possible to keep the country free from infection. It is only upon this condition that the cattle producers of the Argentine Republic can ever hope to regain their market for live cattle in England.

The people of the Argentine Republic have felt that a strong influence in their favor might be created in England if they could only show the English breeders the importance of the Argentine Republic market for pure-bred live-stock. They have done this, and when the English market is again open to them, as it promises soon to be, the influence of the English breeders will be found to have been a most potent factor in accomplishing this end. Therefore, it must be clear that the exclusion of English cattle from the Argentine Republic was not a veterinary matter at all, and was not a thing for which any veterinarian can justly be commended or condemned. It was a piece of broad statesmanship or diplomacy, and the effect has been beneficial to the people of the Argentine Republic.

Why does not the *Breeders' Gazette* blame veterinarians for the exclusion of American and Canadian cattle from the British Isles and the enforcement of the rule that requires them to be slaughtered within ten days upon the docks where they are landed? There has been no contagious pleuropneumonia in the United States for ten years and more. There has been no cattle plague, and there has been no suspicion of foot-and-mouth disease until during the last month. It is well known that this English regulation is not a veterinary or sanitary regulation in any sense. It is merely a subterfuge for protection in a country that is not willing to openly abandon its free-trade policy. The people of Britain have no more right to complain of the exclusion of their cattle from the Argentine Republic on the alleged ground of foot-and-mouth disease than have the people of the United Sitates to complain of the exclusion of their cattle from England on the alleged ground of contagious pleuropneumonia.

It is an historic fact that the *rash, inconsiderate, unjustifiable, injurious* quarantines are those that are established by non-veterinarian authorities. Witness the act of the Governor of Illinois, advised by his lay cattle commissioners and his non-professional, unqualified State veterinary surgeon. It is a miracle that this foolhardy quarantine order against States absolutely uninfected and unsuspected was not the means of entirely killing, for the time at least, the whole export trade in live animals from the United States. If the State of Illinois had a live-stock sanitary board, organized as such boards ought to be, and containing at least one educated veterinarian, such a thing could not possibly have occurred. Nor could many of the other hysterical quarantines established by the cattle authorities of Illinois and Western States have been applied, or even considered, if the respective boards had been properly constituted and contained a veterinary member in a position of responsibility and authority.

It is too much to expect that the *Breeders' Gazette* will admit any of this, and we may confidently predict that that paper will continue to cast slurs upon the veterinary profession and endeavor to cause the impression that it is responsible for every unfortunate circumstance affecting the live-stock of the country. In its last issue, on page 1113, it says: "In view of the fact that foot-and-mouth disease could only have been introduced by importation, it becomes an interesting question as to where these alleged cases originated . . . ; it is difficult to imagine how genuine foot-and-mouth disease could secure entry." Here is an innuendo to the effect that some of the leading veterinarians of the United States who investigated the foot-and-mouth disease situation in New England do not know what they are talking about, and are not able to recognize this disease. There is now a lot of veterinarians in New England who are working day and night with all the energy and enthusiasm that men can command to eradicate as promptly and economically as possible a scourge that threatens the live-stock industry of the whole country, and which, if it should escape to the West, is capable of causing appalling losses. These men are doing infinitely more work than their meagre salary pays for, on account of their professional pride and the desire to make a record in the eradication of this disease that will forever stand to the credit of American veterinary sanitation. The encouragement that they receive from this self-styled organ of the American stock farm is a slur to the effect that they do not know what they are working on.

If the editor of the *Breeders' Gazette* ever has a word of appreciation for the work of the veterinarian upon whose efforts the prosperity of the live-stock industry depends we shall be glad to give him credit for it.

